# Paracetamol prescriptions in older people hospitalized in a French geriatric hospital

**DOI:** 10.1002/hsr2.1565

**Published:** 2023-09-19

**Authors:** Frederic Baud, Nadia Ladjouzi, Jihène Ben Hassen, Nesrine El Omeiri, Hendriniainia Raveloson, Lucile Brondin, Camille Ben Rahal, Cid Ould Ouali, Georges Zouloumis, Joël Schlatter

**Affiliations:** ^1^ Médecine gériatrique aigue, Hôpital Paul Doumer Assistance Publique des Hôpitaux de Paris Labruyère France; ^2^ Service de soins et réadaptation, Hôpital Paul Doumer Assistance Publique des Hôpitaux de Paris Labruyère France; ^3^ Service de long séjour, Hôpital Paul Doumer Assistance Publique des Hôpitaux de Paris Labruyère France; ^4^ Pharmacie, Hôpital Paul Doumer Assistance Publique des Hôpitaux de Paris Labruyère France

**Keywords:** dose, older adult, paracetamol, prescription, reassessment

## 1 BACKGROUND

Paracetamol is one of the most widely used drug in the world for the management of pain or fever, in particular among older people aged 60 years or older.^1^, ^2^ In France, the consumption of the drug is currently the highest in Europe, with an increase of 53% from 2006 to 2015.^3^ As paracetamol was considered safe at therapeutic doses, it has been recommended as a first‐line drug in pain management guidelines, especially for aged and frail patients.^4^ However, the pharmacokinetic of paracetamol may be influenced by physiological and pathological changes in older patients, especially those who are underweight or frail.^2^ Studies have reported a reduction of paracetamol clearance with aging and frailty, resulting in an increase of paracetamol serum levels.^5^, ^6^ Moreover, liver damage caused by paracetamol at high dose can occur particularly into older patients who are malnourished or weigh <50 kg.^7^ One explanation is the increase of the toxic metabolite of paracetamol, *N*‐acetyl‐p‐benzoquinone‐imine, by glutathione depletion.^8^ In addition, the impact of multimorbidity and polypharmacy prevalent in older people on the pharmacokinetics of paracetamol remains to be investigated.^9^ Studies supported the evidence of a clinically significant interaction between warfarin and paracetamol, requiring close monitoring taking the drugs in combination.^10^, ^11^ Some epidemiological studies also suggested that chronic paracetamol use might be associated with renal or cardiovascular disease, gastrointestinal disorders, and asthma, but these findings were inconclusive and had no proven relevance to acute episodic use.^12^, ^13^ However, it remains the analgesic of choice in the older patient and the appropriateness of its continuation should be regularly reassessed including pain evaluation.

Notwithstanding the concern regarding potentially excessive dosing in frail and low‐body‐weight older people in hospital, there are few published data on the subject outlining the prescribing and monitoring practices of paracetamol in these people.

Therefore, the aim of this study was to describe in real practice the paracetamol use in elderly hospitalized patients.

## 2 METHODS

### 2.1 Study design

A retrospective observational study of patients hospitalized into a French geriatric public hospital for oral paracetamol prescriptions was performed between January 2021 and December 2021. Ethical approval from the Ethic Committee of the Paul Doumer Hospital was waived before the start of data collection, as the study was exclusively observational. Inclusion criteria comprised participants aged over 70 years and who were admitted to hospital for more than 7 days in the acute geriatric unit (AGU), care and rehabilitation unit (CRU), and long‐term care unit (LTCU). Exclusion criteria comprised patients who had received paracetamol‐containing combination analgesics and drugs interacting severely or moderately based on the Micromedex database (isoniazid, imatinib, warfarin, phenytoin, acenocoumarol, zidovudine, carbamazepine, and ethanol). The paracetamol combination with analgesic has been excluded from our therapeutic arsenal because of the higher potential for drug overdose. So, we decide arbitrary to exclude this combination.

### 2.2 Data collection

Subjects were identified through a retrospective review of our electronic prescribing and medical records of all patients with oral paracetamol prescription. The physician assigned to analyze and collect medical information extracted data from subjects who are eligible for inclusion. Fully independent of the study, he selected 30 patients for this study. The data gathered information regarding the patient demographics, biochemical measurements, paracetamol administration, related drugs, and the duration of the patients’ stay. In addition, the paracetamol dose received each day, the duration of paracetamol prescription for regular administration or when needed, the cumulative dose, and the reassessment of prescription were also recorded from electronic records. Physicians collected data. Paracetamol dosing was compared with consensus guidelines that recommended a maximum safe dose <4 g/24 h with a dosage adjustment in the context of comorbidities and co‐prescription of anticoagulants in patients over 65–70 years of age.^14^, ^15^ Moreover, some guidelines included warning that some patients may be at the risk of experience toxicity with a body‐weight under 50 kg and suggested a dose reduction when paracetamol is used regularly in these patients.^4^, ^15^, ^16^

### 2.3 Data analysis

Data were provided and analyzed using Microsoft Excel for mean, SD, and extrema.

## 3 RESULTS

The demographic and chemical characteristics of participants are summarized in Table 1. The age ranged from 71 to 97 years of age, with an average of 87.3 (SD ± 6.5). The average length of stay was 60 days (SD ± 73) with range from 7 to 305 days. No participant had an alcoholism addiction or hepatic failure (Table 1). The modification of diet in renal disease was classified to Stage 3 to 5 in 13 patients, equivalent to a moderate decrease to renal failure (Table 1). From the 30 patients, all patients are regarded as frail, including four with a weight of <50 kg. For most patients, the inpatient paracetamol order was for when needed (*n* = 23, 74%) (Table 2). The paracetamol order was not reported in 19% of patients. All patients received a theoretical paracetamol dose under 4 g per day as recommended in consensus guidelines (Table 2). The duration of prescription differed depending in the clinical unit (Table 2). In AGU and CRU, the mean of prescription days was fairly short with 8 (±4, range 1–14) days and 44 (±28, range 9–90) days, respectively. As opposed, in LTCU where the average of length of stay is extensive, the mean of prescription days was high with 824 (±687, range 24–1996) days. The mean daily dose was under 60 mg/kg but with a wide range of 30.7–85.7 mg/kg (Table 2). The patients weighed <50 kg received a paracetamol dose higher than recommended in consensus guidelines (mean daily dose 74.2 ± 8.5 mg/kg, range 62–85.7). The theoretical cumulative paracetamol dosing for patients admitted in AGU, CRU, and LRCU was estimated to 24 ± 12, 125 ± 85, and 2472 ± 2061 g, respectively. However, the cumulative paracetamol dosing actually received was 9 ± 11 (0–42) for AGU, 31 ± 37 (2–146) for CRU, and 614 ± 556 (22–1536) for LCRU (Table 2). This variation could be explained by the paracetamol order in “when needed” and the respect of paracetamol administration by nurses correlated with the pain assessment. In eight patients, the paracetamol prescription was initiated, despite no pain being recorded in the evaluation of the pain (Table 2).

**Table 1:** Demographic information of 30 inpatients.

Characteristics	Mean ± SD (extrema)
Age (years)	87.3 ± 6.5 (71–97)
Sex (male/female)	12/18
Clinical unit	
Critical geriatric medicine unit	12 patients
CRU	12 patients
LTCU	6 patients
Duration of stay (days)	60 ± 73 (7–305)
Weight (kg)	61.7 ± 13.4 (35.0–97.8)
<50 kg	4 patients
>50 kg	26 patients
BMI (kg/m^2^)	23.7 ± 4.7 (14.5–37.1)

Abbreviations: BMI, body mass index; CRU, care and rehabilitation unit; LTCU, long‐term care unit.

**Table 2:** Management of paracetamol prescription and administration.

	AGU (*n* = 12)	CRU (*n* = 12)	LTCU (*n* = 6)
Paracetamol order			
When needed	9 patients (75%)	9 patients (75%)	5 patients (83%)
Without order	2 patient (17%)	2 patients (17%)	1 patients (29%)
For regular administration	1 patient (8%)	1 patients (8%)	0 patient
Duration of prescription (days)	8 ± 4 (1–14)	44 ± 28 (9–90)	824 ± 687 (24–1996)
Paracetamol dosing	1 g × 3/day	1 g × 3/day	1 g × 3/day
Paracetamol dosing in mg/kg (*n* = 30)	57.0 ± 17.6 (30.7–85.7)	51 ± 8 (35–71)	47 ± 7 (36–58)
Paracetamol dosing in mg/kg for patients <50 kg (*n* = 4)	74.2 ± 8.5 (62–85.7)	–	–
Theoretical cumulative paracetamol dosing (g)	24 ± 12 (3–42)	125 ± 85 (27–270)	2472 ± 2061(72–5988)
Cumulative paracetamol dosing actually received	9 ± 11 (0–42)	31 ± 37 (2–146)	614 ± 556 (22–1536)
Visual analog scale at day 0 of prescription	0 (*n* = 2), 1 (*n* = 1), 2 (*n* = 7), 3 (*n* = 2)	0 (*n* = 5), 2 (*n* = 7)	0 (*n* = 1), 1 (*n* = 2), 2 (*n* = 2), 3 (*n* = 1)=
Number of combined medicines	8 ± 3 (4–13)	9 ± 4 (3–18)	8 ± 4 (2–12)

Abbreviation: AGU, acute geriatric unit.

## 4 DISCUSSION

The management of pain in the older population varies from that of younger people. Indeed, the clinical expressions of pain are often complex and multifactorial in the elderly population. In addition, older people may underreport pain particularly for patients with cognitive disorders. Although changes with normal aging can affect disposition, metabolism, and responses to analgesic medications.^14^ As paracetamol is not associated with significant adverse effects than traditional nonsteroidal anti‐inflammatory drugs, paracetamol is recommended as first‐line therapy for the management of pain in older adults. Likewise, some studies reported paracetamol regimens in older adults that involved administrations >3 or 4 g/d.^2^, ^17^, ^18^ In a French study, 50% of patients aged >75 years with risk factor for toxicity (low body weight or malnutrition) had their paracetamol dose not reduced.^17^ In a recent Swiss study, the maximal daily dose of 60 mg/kg was exceeded in 30.1% of patients weighing <50 kg, as well as in 42.3% of patients weighing <60 kg.^18^

In our hospital, paracetamol was the first drug taken in units. The paracetamol order was mainly in “when needed” without time prescription restriction in LTCU. However, the paracetamol dosing was systematically 1 g × 3 per day regardless of the patient's weight or frailty. In 19% of patients, the paracetamol dose was higher (range 62–85.7 mg/kg/d) than recommended by consensus guidelines, in particular for patients in AGU. Therefore, admission in different ward unit might affect the management of paracetamol. This situation may indicate insufficient clinician awareness of guidelines for paracetamol use in the frail and underweight older population. Nevertheless, the reasons why paracetamol doses were not adjusted particularly to low‐weight or frailty older population were not investigated. One contributing factor could be the absence of local guidelines for the paracetamol prescription in the elderly. Another explanation may be the lack of decision support for paracetamol dosing integrated into the electronic medication system. The implementation of an electronic tool with alerts for excessive paracetamol in a Swiss hospital reported >70% of excessive dosing and permitted pharmaceutical interventions.^18^ At the study hospital, it has been proposed setting up a protocol for prescribing paracetamol at 1 g three times a day for the oral route and 15 mg/kg/d for the intravenous route. Moreover, support on the correlation between pain assessment performed by nurses and paracetamol use was provided to ensure proper administration of the drug. For the low‐weight and frailty patients, pharmacists proposed by electronic prescribing system to adapt the paracetamol dosing to maximum 50 mg/kg/d.

## 5 CONCLUSION

Patients were prescribed paracetamol doses that were rarely adapted to the weight or frailty of older population, and for a prolonged period into LTCU. The use of an automatic electronic alert system for paracetamol prescriptions and cumulative daily dose may reduce the risk of overdose.

## AUTHOR CONTRIBUTIONS

**Frederic Baud**: Conceptualization; data curation; formal analysis; funding acquisition; methodology; resources; supervision. **Nadia Ladjouzi**: Validation; visualization. **Jihène Ben Hassen**: Validation. **Nesrine El Omeiri**: Validation. **Hendriniainia Raveloson**: Validation. **Lucile Brondin**: Visualization. **Camille Ben Rahal**: Visualization. **Cid Ould Ouali**: Validation; visualization. **Georges Zouloumis**: Validation; visualization. **Joël Schlatter**: Conceptualization; data curation; formal analysis; funding acquisition; methodology; validation; writing—original draft. All authors have read and approved the final version of the manuscript. Corresponding author had full access to all of the data in this study and takes complete responsibility for the integrity of the data and the accuracy of the data analysis.

## CONFLICT OF INTEREST STATEMENT

The authors declare no conflict of interest.

## ETHICS STATEMENT

The requirement for informed consent was waived by the Ethic Committee of the Paul Doumer Hospital because of the retrospective nature of the study.

## TRANSPARENCY STATEMENT

The lead author Joël Schlatter affirms that this manuscript is an honest, accurate, and transparent account of the study being reported; that no important aspects of the study have been omitted; and that any discrepancies from the study as planned (and, if relevant, registered) have been explained.

## Data Availability

All relevant data have been presented in the manuscript and further inquiry can be directed to the corresponding author.
